# Cervical Spine Range of Motion Reliability with Two Methods and Associations with Demographics, Forward Head Posture, and Respiratory Mechanics in Patients with Non-Specific Chronic Neck Pain

**DOI:** 10.3390/jfmk10030269

**Published:** 2025-07-16

**Authors:** Petros I. Tatsios, Eirini Grammatopoulou, Zacharias Dimitriadis, Irini Patsaki, George Gioftsos, George A. Koumantakis

**Affiliations:** 1 Laboratory of Advanced Physiotherapy, Physiotherapy Department, School of Health & Care Sciences, University of West Attica, 12243 Athens, Greece; ptatsios@uniwa.gr (P.I.T.); igrammat@uniwa.gr (E.G.); ipatsaki@uniwa.gr (I.P.); gioftsos@uniwa.gr (G.G.); 2 Health Assessment & Quality of Life Laboratory, Physiotherapy Department, University of Thessaly, 35100 Lamia, Greece; zdimitriadis@uth.gr

**Keywords:** musculoskeletal, pain, biomechanics, mHealth, dysfunctional breathing

## Abstract

**Objectives**: New smartphone-based methods for measuring cervical spine range of motion (CS-ROM) and posture are emerging. The purpose of this study was to assess the reliability and validity of three such methods in patients with non-specific chronic neck pain (NSCNP). **Methods**: The within-day test–retest reliability of CS-ROM and forward head posture (craniovertebral angle-CVA) was examined in 45 patients with NSCNP. CS-ROM was simultaneously measured with an accelerometer sensor (KFORCE Sens^®^) and a mobile phone device (iHandy and Compass apps), testing the accuracy of each and the parallel-forms reliability between the two methods. For construct validity, correlations of CS-ROM with demographics, lifestyle, and other cervical and thoracic spine biomechanically based measures were examined in 90 patients with NSCNP. Male–female differences were also explored. **Results**: Both methods were reliable, with measurements concurring between the two devices in all six movement directions (intraclass correlation coefficient/ICC = 0.90–0.99, standard error of the measurement/SEM = 0.54–3.09°). Male–female differences were only noted for two CS-ROM measures and CVA. Significant associations were documented: (a) between the six CS-ROM measures (R = 0.22–0.54, *p* < 0.05), (b) participants’ age with five out of six CS-ROM measures (R = 0.23–0.40, *p* < 0.05) and CVA (R = 0.21, *p* < 0.05), (c) CVA with two out of six CS-ROM measures (extension R = 0.29, *p* = 0.005 and left-side flexion R = 0.21, *p* < 0.05), body mass (R = −0.39, *p* < 0.001), body mass index (R = −0.52, *p* < 0.001), and chest wall expansion (R = 0.24–0.29, *p* < 0.05). Significantly lower forward head posture was noted in subjects with a high level of physical activity relative to those with a low level of physical activity. **Conclusions**: The reliability of both CS-ROM methods was excellent. Reductions in CS-ROM and increases in CVA were age-dependent in NSCNP. The significant relationship identified between CVA and CWE possibly signifies interconnections between NSCNP and the biomechanical aspect of dysfunctional breathing.

## 1. Introduction

Non-specific chronic neck pain (NSCNP) is a significant and multifaceted health problem in today’s society, ranking as the fourth leading cause of disability, with an annual prevalence rate exceeding 30% [1]. The economic burden of neck pain is considerable, encompassing treatment costs, decreased productivity, and job-related issues. Globally, in 2017, the age-standardized prevalence and incidence rates of neck pain were 3551.1 and 806.6 per 100,000, respectively, and the years lived with disability from neck pain per 100,000 population was 352.0 (245.6 to 493.3) [2].

NSCNP is defined as neck pain that lasts for more than 12 weeks, even after the injury has healed, or that recurs intermittently over a prolonged period [3]. In patients with NSCNP, studies have identified symptoms such as local hyperalgesia; impaired conditioned pain modulation; psychological disturbances, including depressive symptoms; pain catastrophizing [4]; reduced neck muscle strength and endurance [5]; alterations in the timing and activation of the cervical muscles; fatty degeneration [6]; and increased forward head posture (FHP) [7]. Additionally, moderate respiratory dysfunction [8] including chest wall expansion [9] and alterations in normal breathing patterns [8] have been noted in this population. Their neck movements may also be limited in quantity and quality [6].

The spine is a multi-segmental kinetic biomechanical chain, and usually, dysfunction in one of its parts may affect an adjacent part. Given the biomechanical link between the cervical and thoracic spine, it is plausible that impaired thoracic spine mobility may contribute to the development of neck disorders [10,11,12]. Kinematic and electromyographic results indicate that motor changes are not isolated to the neck and also affect the thoracic spine, thus highlighting the need to assess the spine’s kinematic variables (position, velocities, and accelerations) of its adjacent segments, which reflect alterations in muscle recruitment [13].

Conversely, neck pain may lead to thoracic spine and rib-cage biomechanical alterations, contributing to dysfunctional breathing [14,15,16], which is multi-dimensional and characterized by biochemical, biomechanical, and psychophysiological dimensions [17].

There is no single definitive treatment for neck pain, with a significant number of patients with NSCNP seeking physical therapy treatment [18]. Physical therapists often conduct thorough physical examinations to assess impairments and effectively monitor the progress of rehabilitation interventions. The American Physical Therapy Association (APTA) guidelines emphasize the importance of using reliable measures to assess changes in a patient’s level of function during treatment for neck pain [19]. One such measure specifically recommended by the APTA is the cervical spine range of motion (CS-ROM) [19,20]. A visual estimation of range of motion (ROM) has been shown to be inaccurate and is not recommended for evaluating passive or active ROM [21].

The evaluation of CS-ROM serves as a typical clinical approach to appraise and categorize individuals experiencing neck pain, determine any functional restrictions, and supply helpful predictive data. Still, clinicians ought to be careful about forming clinical opinions mainly on CS-ROM [22]. An analysis of chronic neck-pain recovery predictors revealed that higher anxiety and limited lateral flexion range of motion were associated with a greater probability of positive results from manual therapy delivered according to the Mulligan concept [23].

A recent systematic review with a meta-analysis [24] mentioned that smartphone apps (clinometer apps, compass apps, and other type of apps) have proven to be reliable tools for measuring CS-ROM in people both with and without neck pain. For physiotherapists assessing neck movement, smartphone applications (apps) offer a valuable resource. However, the overall quality of the research supporting these apps is still limited. More rigorous studies with larger sample sizes are necessary to strengthen the evidence base for using smartphone apps to assess CS-ROM.

The purpose of this study was to evaluate the measurement properties of an accelerometer sensor vs. a mobile phone device as measures of CS-ROM. Specifically, we sought to assess their within-day test–retest reliability and the parallel-forms reliability of the two measurement methods, as well as the within-day test–retest reliability of FHP. Furthermore, the association of CS-ROM with demographics, FHP, and other biomechanical respiratory measures, and the male–female differences in those measures, were examined.

## 2. Materials and Methods

### 2.1. Sample

A non-random sample of 90 participants, aged between 25 and 65 years, was recruited for this study on a voluntary basis. The study was conducted from June 2022 to July 2023. These individuals had been diagnosed with NSCNP, defined as pain originating anywhere in the neck, from the occiput to the top of the scapula, extending laterally to the top of the shoulder and the lateral end of the clavicle, but excluding the shoulder joint and resulting solely from injury to the spinal soft tissues.

Patients were included in the study if they met the following criteria: persistent mechanical neck pain for more than 3 months (chronic) [25], neck pain of grade I or II according to the Task Force on Neck Pain grading [26], negative special tests (Spurling’s test, traction test, Upper Limb Tension Test, and shoulder abduction test), and the absence of any other significant comorbidities. Patients were excluded from the study if they presented with signs and symptoms of cervical radiculopathy, neck pain attributed to vertigo or a previous whiplash injury, a previous fracture or surgery of the spine, neurological or inflammatory spinal cord disease or inflammatory arthritis, dementia, respiratory or cardiac failure, asthma or other respiratory pathologies, infectious diseases or neoplasms, fibromyalgia, metabolic diseases, and osteoporosis, pregnancy, or psychological disorders. Additionally, patients with red flags, such as nocturnal pain, severe muscle spasms, unexplained weight loss, feelings of fatigue, daily headaches, dyspnea, tachypnea, confusion, or loss of consciousness, were also excluded.

### 2.2. Ethics

Ethical approval was obtained by the Ethics Committee of the University of West Attica, Greece (51758—01/06/2022) for this study, which was conducted according to the Declaration of Helsinki stipulations.

### 2.3. Study Design

This study was cross-sectional and comprised two parts: (a) the within-device test–retest and between-devices same-day reliability of CS-ROM and of FHP, and (b) the construct validity of CS-ROM via associations with patient demographics, and biomechanical measures relating to CS posture and respiratory mechanics in patients with NSCNP, as well as the between-gender differences of those measures.

### 2.4. Procedures

All participants completed a written questionnaire on demographic information, lifestyle factors (leisure-time physical activity and smoking frequency), and their symptoms’ duration. All participants underwent a standardized assessment, including a detailed medical history and a battery of specialized orthopedic tests (Spurling’s test, traction test, upper limb tension test, and shoulder abduction test).

One rater (A.T.) with more than 20 years’ experience as a physical therapist performed all measurements, following a standardized measurement protocol.

#### 2.4.1. Cervical Spine Range of Motion

**Figure 1:**
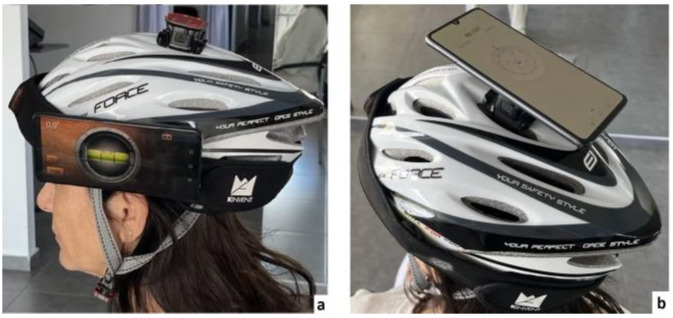
Details of placement of both devices on a helmet fastened securely to the participants’ heads. Placement of mobile phone to the side (**a**), and on top (**b**) of the helmet.

The CS-ROM was measured with an accelerometer sensor (KFORCE SENS^®^, KINVENT, Montpellier, France), a small (15 × 56 × 35 mm), lightweight (40 g) inertial sensor-based electro goniometer. Concurrently, CS-ROM was measured with a mobile phone device (Model P30/ELE-L29, Huawei Smartphone, Shenzhen, China 2019, purchased January 2021), utilizing two accelerometer, gyroscopic, and magnetometer-based apps: the iHandy Level: https://play.google.com/store/apps/details?id=com.ihandysoft.carpenter.level.free&hl=et&gl=US (accessed on 10 May 2022) and the pre-installed Compass app of the device (https://consumer.huawei.com/uk/support/article/en-gb15758616/, accessed on 10 May 2022). Both devices were placed on a helmet fastened securely to the participants’ heads (Figure 1), to determine the accuracy of each device as well as the parallel-forms reliability between the two devices in measuring CS-ROM. The helmet was fitted securely using the helmet’s adjustable chin straps, and for hygiene purposes, each participant wore a disposable surgical cap (made of non-woven material) with an elastic band to prevent direct contact between the head and the helmet. The accelerometer sensor of the KFORCE SENS^®^ device was placed inside a pocket of a strap that was securely fastened around the helmet. A stable base was attached to the top of the helmet using straps (for the assessment of right and left rotation), and the upper surface of the base was magnetic. A magnet was also attached to the back of the helmet (for the assessment of left- and right-side flexion), as well as to the side of the helmet (for the assessment of flexion-extension) and the back surface of the smartphone to ensure the accurate and stable placement of the mobile device. Both devices simultaneously measured each of the movements studied.

Specifically, clinometer apps (similar to the iHandy Level app) showed reliable and valid measurements for frontal- and sagittal-plane CS-ROM in people with and without neck pain. Additionally, compass apps provided valid and reliable assessments of horizontal cervical range of motion in a seated posture [24,27].

**Figure 2:**
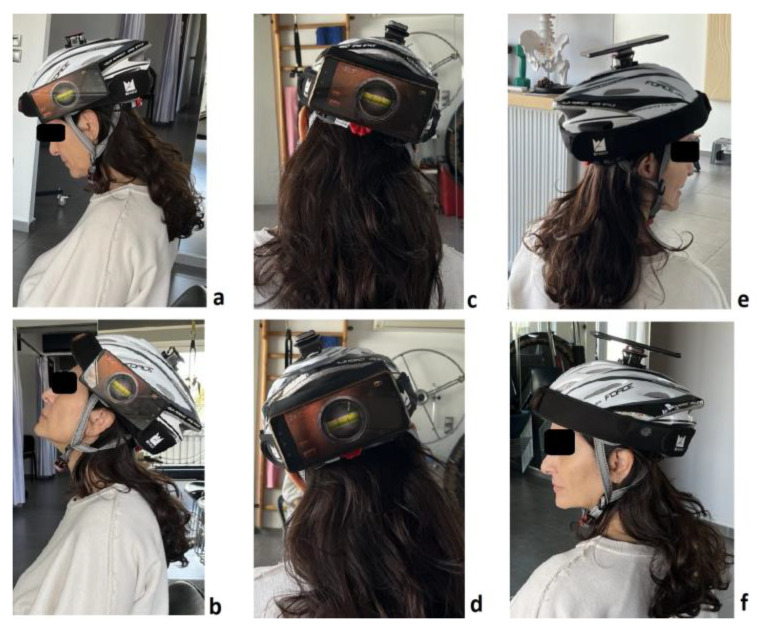
Measurements of cervical spine ROM in flexion (**a**), extension (**b**), right- and left-side flexion (**c**,**d**), and right and left rotation (**e**,**f**).

All measurements were performed while participants were sitting, and the chair used was stationary (without wheels) and had a backrest. All measurements were taken from the same seated position. Participants sat in a relaxed position, not leaning against the backrest, with their palms on their thighs and feet flat on the floor during the measurements. The CS-ROM assessment was conducted in the following order for all participants: flexion, extension, right lateral flexion, left lateral flexion, right rotation, and left rotation (Figure 2).

Additionally, the accelerometer sensor of the KFORCE SENS^®^ device was calibrated at the start of each different movement. Three measures were taken for each movement, and the average was used to determine the CS-ROM per direction of movement. The time allowed for each of the three measures per direction of movement was 5 s, with a 5 s rest period between them. A second set of three measures was taken one hour later. An earlier study indicated excellent CS-ROM reliability within a single testing session using the KFORCE SENS^®^ [28].

#### 2.4.2. Forward Head Posture

Forward head posture (FHP) is characterized by the head being in a forward position relative to the neck in the sagittal plane [29]. The FHP measurement was conducted via lateral photography, according to one of the four methods presented in a previous study [29]. A Huawei P30 ELE-L29 smartphone with a resolution of 2340 × 1080 and a tripod (Rollei Smartphone Tripod Traveler, Hamburg, Germany, purchased March 2022) were used to capture the images. Other materials used included a colored marker to mark the location of the seventh cervical vertebra.

**Figure 3:**
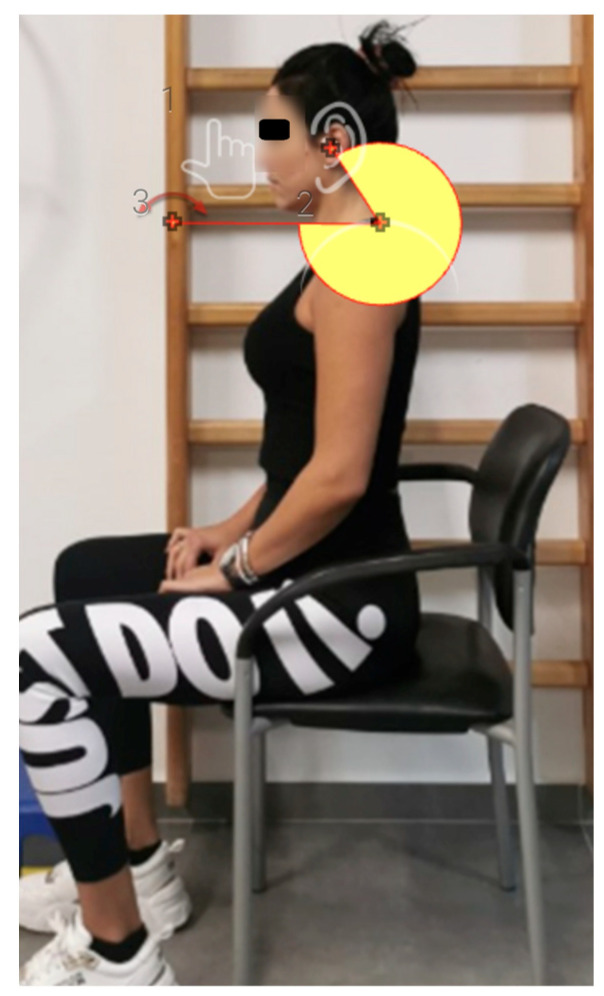
Measurement of craniovertebral (CVA) angle with the Forward Head Posture (FHP) app.

The craniovertebral angle (CVA) is the angle formed by the line connecting C7 to the tragus of the ear and a line parallel to the horizontal plane passing through the C7 marker. During the initial visit, the seventh cervical vertebra (C7) was palpated, and a marker was placed on the skin surface over the tip of its spinous process. Subsequently, the patients were asked to sit relaxed in their natural sitting position, with their feet together, touching the ground, and their hands resting on their legs. They were also asked to look at a specific point at eye level. The examiner adjusted the tripod and smartphone so that each participant’s head and cervical spine were clearly depicted. The camera was positioned 3 m away from the patient, and three lateral photographs were taken. Participants were not allowed to lean against the backrest of the chair or lean their body forward in a relaxed position [29]. The ‘Forward Head Posture-FHP’ app was used to calculate the CVA (https://play.google.com/store/apps/details?id=com.ysjworld.fhp&hl=en_GB, accessed on 10 May 2022). Additionally, the image for CVA calculation (numbers 1–3, red crosses) were appropriately processed by blurring the facial features of the participants to ensure anonymity (Figure 3).

#### 2.4.3. Chest Wall Expansion

Moll’s 1972 description of chest wall expansion (CWE) measurement has since been widely applied in assessing various conditions, such as ankylosing spondylitis, asthma, COPD, and thoracic scoliosis [30,31]. Chest wall expansion has also been used to evaluate abnormal breathing patterns, contributing to the biomechanical dimension of DB, and the efficacy of physical therapies like respiratory muscle training and release [30]. The extent of CWE dictates lung volume and functional ability. A recent study indicated a positive correlation between respiratory muscles’ strength and CWE, diaphragmatic excursion, and functional capacity in healthy subjects [32].

The measurement was conducted using a 1 m long inelastic tape measure, divided into centimeters (cm). The tape was placed at the height of the axilla for the upper chest expansion (CWE-Up), and then at the level of the xiphoid process for lower chest expansion (CWE-Lw). Measurements were taken at maximum inspiration and maximum expiration. The reliability of this method has been previously verified [9]. The examiner secured the zero point of the measuring tape to the spinous process of the vertebra. The tape was then held in place by the examiner’s index finger, ensuring no extra pressure was applied to the subject’s body [30].

#### 2.4.4. Hi-Lo Breathing Pattern Assessment

This test evaluates biomechanical dysfunctional breathing (DB) [16]. Upper chest breathing, also known as apical breathing, is the most common dysfunctional breathing pattern seen at rest. It is defined by a dominant expansion of the upper chest during the inspiratory phase. Paradoxical breathing is another biomechanical breathing dysfunction, where the typical outward movement of the lower abdomen during inhalation is reversed, resulting in an inward motion [33]. The Hi-Lo breathing pattern classification is a manual assessment to determine upper and lower rib-cage/abdominal movement, to identify respiratory rate, rhythm, the degree of movement, and the timing relationship between the upper and lower respiratory regions [34].

The examiner stands in front and slightly to the side of the individual, placing one hand on the patient’s sternum and the other on their upper abdomen. Whether chest or abdominal movement is more prominent during breathing and to what extent is then assessed. The examiner also checks for paradoxical breathing. The dominance of thoracic or abdominal breathing is scored between one and three [35].

Previous studies have indicated acceptable reliability and validity for the Hi-Lo test [16,36,37]. In a previous study [16], an 88% agreement was observed among examiners, with a Kappa value of 0.75, based on the assessment of 43 participants. An investigation conducted by Courtney et al., encompassing 56 osteopaths and osteopathic students who received instruction in the Manual Assessment of Respiratory Motion (MARM) and the Hi-Lo Breathing Assessment, coupled with training in the simulation of breathing patterns, provides evidence supporting the validity of the Hi-Lo assessment method [36].

#### 2.4.5. Respiratory Rate

Respiratory rate (RR), expressed in breaths per minute, was measured, and the data was obtained directly from a capnography unit’s output (Nonin’s LifeSense^®^ II, Nonin Medical Inc., Plymouth, MN, USA). Studies have shown that an increased RR (>18) is often associated with dysfunctional breathing (DB) [33]. Typically, the respiratory rate is under 16 breaths per minute, and for an average-sized individual, their tidal volume is less than 600 mL at quiet rest, with effortless ease [38,39,40,41].

### 2.5. Statistical Analysis

Descriptive statistics of continuous variables were presented in detail, according to the distribution of each variable. The normality of the distribution of continuous variables (demographics, CS-ROM, CVA, RR, and CWE) was analyzed with the Kolmogorov–Smirnov test.

To assess whether age, height, weight, and BMI had an effect on the patients’ biomechanical-related variables (CS-ROM, CVA, RR, and CWE), appropriate correlations were employed, and to assess the effect of gender and of the breathing pattern (Hi-Lo) on those variables, the independent samples t-test was used. Also, the effect of lifestyle factors (smoking frequency and leisure-time physical activity) on the biomechanical-related variables and respiratory variables was examined with the chi-square test.

The within-day test–retest reliability of CS-ROM for each device was separately tested for each device with the two-way random-effects absolute-agreement multiple-measurements intraclass correlation coefficient (ICC_2,3_). The between-devices parallel-forms reliability [42] was calculated using the two-way random-effects absolute-agreement single-measurement intraclass correlation coefficient (ICC_2,1_) [43], the standard error of the measurement (SEM), and the minimum detectable change (MDC_95%_) [44,45]. The within-day test–retest reliability of CVA was calculated using the two-way random-effects absolute-agreement single-measurement intraclass correlation coefficient (ICC_2,1_) [43], the standard error of the measurement (SEM), and the minimum detectable change (MDC_95%_) [44,45]. ICC values were considered as poor if lower than 0.5, while between 0.5 and 0.75 was considered moderate, between 0.75 and 0.90 was considered good, and greater than 0.90 was considered excellent [43]. The SEM and MDC_95%_ represent the measurement error level in the same values as the original measurement (degrees), with the MDC corresponding to the smallest detectable amount of change that cannot be attributed to measurement error [44,45].

The construct validity of the CS-ROM was further examined via correlations with patients’ biomechanical-related variables (CVA, RR, and CWE) [46]. Correlations were classified as negligible (0.0–0.25), fair (0.25–0.50), moderate to good (0.50–0.75), and good to excellent (>0.75) [45].

The minimum sample size required for the study was estimated taking into account the large number of correlations and adjusting the level of statistical significance based on the Holm–Bonferroni method [47]. Therefore, the minimum sample size for conducting 24 correlations of primary interest (six CS-ROM measures × three biomechanics-related variables), with an adjusted statistical significance level α = 0.05/18 = 0.0028, to achieve 90% statistical power with a moderate correlation coefficient r = 0.50, was calculated to be *n* = 63 participants, calculated with a relevant algorithm for correlational studies (https://sample-size.net/correlation-sample-size/, accessed on 10 May 2022) [48]. All other analyses were performed with the IBM SPSS Statistics v.29.02.00 software.

## 3. Results

### 3.1. Demographics

Overall, 90 patients (54 women) with NSCNP that were referred for physical therapy at a private practice participated in this study. The majority of continuous variables (10 out of 14) were normally distributed (*p* > 0.05), apart from height, BMI, CWE-Up, and CWE-Lw; therefore, descriptive data were presented as mean (SD), maximum, and minimum statistics for all continuous variables and analyzed with parametric statistics. Participants’ continuous demographic characteristics are displayed in Table 1. Each patient had a current pain episode that lasted longer than three months, with a mean (SD) of 6.39 (1.72) months. Participants’ categorical demographic characteristics are displayed in Table 2.

**Table 1:** Descriptive statistics of participants’ demographic characteristics and pain duration (*n* = 90).

	Mean (SD)	Min–Max
Age (y)	41.25 (10.93)	24–69
Height (m)	1.72 (0.08)	1.60–1.90
Body Mass (kg)	75.25 (15.63)	48–120
Body Mass Index (kg/m^2^)	25.39 (4.28)	18.34–35.99
Pain Duration (months)	6.39 (1.72)	4–11

y: years, kg: kilograms, m: meters.

**Table 2:** Categorical descriptive statistics of participants’ demographic and lifestyle characteristics (*n* = 90).

	*n*
Sex	
Male	36
Female	54
Marital Status	
Single	29
Married	49
Divorced/Widower	12
Education level	
Secondary	44
Higher	46
Smoking frequency	
No	60
Sometimes	18
Often	12
Leisure-Time Physical Activity	
No	0
Low	36
Moderate	37
High	17

The descriptive statistics of participants’ CS-ROM, CVA, and the respiratory biomechanical-related measures (total and per gender) are displayed in Table 3. Only flexion, L rotation, and the CVA were significantly affected by gender (3 out of 10 variables).

In addition, the descriptive statistics of participants’ CS-ROM and three key demographic variables, as well as of the respiratory biomechanical-related measures classified according to the breathing pattern, are displayed in Table 4. Also, the effect of self-reported leisure-time physical activity and smoking frequency on breathing pattern was examined. Only the extension CS-ROM, the RR, and the CWE-Up were significantly influenced by the breathing pattern (3 out of 14 variables).

### 3.2. Same-Day Reliability of CS-ROM (Within and Between Devices)

The reliability level of the mean of three sequential CS-ROM measures taken in each movement direction on the same day, with a 1 h difference between test and retest by the same examiner, was evaluated. The within-devices ICC_2,3_ values were excellent in general both for the KFORCE SENS^®^ (0.97–0.98) and the mobile apps (0.90–0.99) for all movement directions. The SEM values ranged between 1.33 and 1.88°, and the MDC_95%_ was between 3.68 and 5.21°, for the KFORCE SENS^®^. For the mobile apps, the SEM values ranged between 1.02 and 3.09°, and the MDC_95%_ was between 2.82 and 8.56°, generally registering an acceptable trial-to-trial error level; however, this was slightly lower for the KFORCE SENS^®^ (Table 5). For the between-devices reliability indices, the ICC_2,1_ values were excellent (0.97–0.99), with the SEM values ranging between 0.54 and 1.74° and the MDC_95%_ between 1.49 and 4.83°, registering an acceptable between-devices error level (Table 5).

### 3.3. Reliability of CVA

The test–retest ICC_2,1_ for CVA was excellent (0.97), with an SEM of 1.39° and an MDC_95%_ of 3.85°, also registering an acceptable trial-to-trial error level (Table 6).

### 3.4. Construct Validity of CS-ROM Measurements

Since both devices were accurate and measurements concurred between the two in all movement directions (flexion, extension, R-/L-side flexion, and R/L rotation), the device that was easier to apply, which was also the one demonstrating the least overall test–retest error, was selected to proceed with measuring CS-ROM for the rest of the participants with NSCNP.

Significant associations (Pearson’s correlations) were documented: (a) between the six CS-ROM measures (R = 0.22–0.54, *p* < 0.05), (b) participants’ age with five out of six CS-ROM measures (R = 0.23–0.40, *p* < 0.05) and CVA (R = 0.21, *p* < 0.05), (c) CVA with two out of six CS-ROM measures (extension R = 0.29, *p* = 0.005 and left-side flexion R = 0.21, *p* < 0.05), body mass (R = −0.39, *p* < 0.001), body mass index (R = −0.52, *p* < 0.001), and chest wall expansion (R = 0.24–0.29, *p* < 0.05). All associations are displayed in Table 7.

There was no effect of the self-reported level of participants’ leisure-time physical activity and all CS-ROM variables. However, there was a significant effect between performing leisure-time physical activity and the CVA (F (2, 87) = 3.67, *p* = 0.029); post hoc tests revealed a statistically significant effect between the CVA of subjects with a low level of activity (M[SD] = 45.46° [9.28]) and those with a high level of activity (M[SD] = 51.45° [6.71]), recording a between-group mean difference (MD) [95% CI] of −5.99° [−11.45 to −0.50]).

## 4. Discussion

The first part of this study was a reliability investigation, examining the within-day test-retest and parallel forms reliability of the KForce Sens^®^ electrogoniometer and the smartphone-based i-Handy Level app and the pre-installed Compass app. The reliability of the CVA measurement was also examined with the method of lateral photography, using the FHP app. No statistically significant differences were found within and between the measurements of the two devices, with a low error level for CS-ROM. The error level was slightly lower for the KFORCE SENS^®^ electrogoniometer in the current study; therefore, measurements for the whole cohort were conducted with this device. Excellent within-day reliability was also reported for CS-ROM measurement with the KFORCE SENS^®^ (ICC = 0.99 and SEM = 1.20–2.28 %) in a group of healthy young participants [28]. In agreement with this study, several studies indicate that smartphone apps, such as the "compass" [49,50,51] and the "clinometer" [49,52], constitute an accessible, economical, and practical method and possess satisfactory reliability and validity for the quantification of CS-ROM in the frontal, transverse and sagittal planes within clinical settings, when compared with gravitational inclinometers.

In contrast to our results, a previous study concluded that clinical assessments of head posture and cervical mobility in adults with neck pain and associated conditions (grades I–III) do not have adequate evidence to support their reliability and validity [53]. Another previous investigation conducted in patients with NP, although reporting a high degree of intra-rater reliability for the Android-based clinometer in the assessment of all cervical movements, found a notable exception of rotation, which exhibited poor to moderate level of reliability [49]. In another study that quantified CS-ROM in a cohort of healthy individuals [54], the Android clinometer application demonstrated excellent intra-rater reliability for cervical flexion, extension, and lateral flexion (ICC = 0.82–0.90), but it was poor for R and L rotation (ICC = 0.05–0.33)—a finding that partly aligns with the results of our present study.

In a study utilizing the KForce Sens^®^ electrogoniometer in 60 healthy adults aged 19–24 years, females had significantly higher CS-ROM in extension and lateral flexion than males (*p* < 0.05) [28]. Conversely, our research revealed that females with NSCNP demonstrated greater CS-ROM in flexion and left rotation compared to males (*p* < 0.05). The difference in study populations between the two studies is most likely responsible for the differences noted. We chose to use only three repetitions for each test direction to reduce patient assessment time, thus lowering the chances of them becoming tired or being in pain, which is in line with the two earlier studies that used the KForce Sens^®^ electrogoniometer for CS-ROM assessment [28,55]. This contrasts with earlier research that advocated for two [56], five [49], or six [57] repetitions per test direction as the benchmark for optimal data acquisition. The difference between the current study and the one by Batatolis et al. 2023 [28] is that, in the latter, the highest of the three measurements was selected, whereas in this study, the average of the three was taken to determine the CS-ROM per movement direction. Differences also existed in the measurement procedure, such as the application of a belt to stabilize the thoracic spine, which was not used in this and our previous study [55], as well as the fact that participants were not leaning against the backrest.

Further differences can be observed between the previous reliability and validity studies of smartphone apps, such as the ‘compass’ and ‘clinometer’ in the measurement method. In most studies, examiners appear to touch and hold the mobile phone in contact with participants’ heads, and the neutral head position was visually adjusted by the examiners before the initiation of each set of measurements [49,50,58]. However, this methodological approach may have introduced measurement inaccuracies stemming from inconsistencies in the initial positioning across all experimental trials. The study by Saptute et al. 2019 [51] is an exception, as they used elasticated Velcro straps to secure the mobile phone to the participants’ heads. However, in that specific study, the researchers only evaluated rotation in the transverse plane. Conversely, in our study, the mobile phone was firmly attached to a magnet on the side (for sagittal-plane measurement), the back (for frontal-plane measurement), or the top (for transverse-plane measurement) of a helmet that was fitted tightly on participants’ heads.

The excellent reliability of the CVA measurement with low measurement error concurs with several previous studies conducted with the lateral photography method [29,59,60]. The CVA measurement in previous studies was either conducted with the Auto CAD [29], the Surgimap software [61,62], or with the FHP app [60], the latter also being used in our study.

Our study further confirms the correlation between age and CS-ROM in five (except for flexion) out of the six movement directions (Table 6) but not with the other demographic characteristics (height, weight, BMI) or with leisure-time physical activity, as measured with a validated brief scale [63]. In accordance with our study, Liu et al. 2015 mention that the regression coefficient for age was determined to be −6.46, indicating an inverse relationship whereby the total ROM decreased by 6.46° for each subsequent decade of life [64]. Specifically, the mean (SD) total ROM in the third decade was 81.49 (14.27)°, which diminished to 53.37 (12.86)° by the eighth decade. Also, Park et al. 2021 mention that age was related only to cervical lateral flexion on both sides (R side: r = −0.423, *p* < 0.05; L side: −0.448, *p* < 0.05) [65], and Quek et al. 2013 [56] mentioned that increased age (60–78 years) was associated with reduced total (Spearman’s R = −0.42, *p* < 0.01) and upper (Spearman R = −0.51, *p* < 0.01) cervical rotation ROM. There was also a significant effect between self-reported leisure-time physical activity [63] and the CVA, specifically between the CVA of subjects reporting a low level of physical activity and those reporting a high level of physical activity (between-group mean difference (MD) [95% CI] of −5.99° [−11.45 to −0.50]). A similar finding has been previously reported by a research study in healthy young adults with a between-group mean difference of 3.57° [66]; however, it had not reached statistical significance, possibly because in that study subjects were younger and the CVA was measured in standing.

The second aim of our study was to examine the associations of aspects of the biomechanical dimension of DB (CWE, Hi-Lo Breathing Assessment, RR) with biomechanical measures of NSCNP, such as CVA, and CS-ROM.

Most participants (81 out of 90) were classified in the Hi group based on their breathing pattern (Table 4), possibly because our study sample consisted of patients with chronic neck pain. The overwhelming majority of participants classified as having the Hi rather than the Lo breathing pattern has been previously reported [16,66,67]. Consequently, it appears physiologically plausible that these patients had adopted an apical breathing pattern (Hi). This interpretation is further supported by many observational studies [16,41,67,68].

Additionally, a statistically significant difference was observed between participants with a different breathing pattern, specifically those exhibiting a Hi breathing pattern having a lower CWE-Up. This finding is perplexing, as one might have expected participants with a Hi respiratory pattern to have a higher CWE-Up. A potential explanation for this observation, other than being a ‘statistical anomaly’, could be that CWE is assessed during maximal inhalation and exhalation, whereas the Hi-Lo assessment evaluates the participants’ breathing pattern during quiet breathing. Furthermore, the age, sex, and BMI of participants did not differ between participants with Hi and Lo breathing patterns (Table 4); therefore, these variables might not have contributed to this finding. In addition, participants with a Hi breathing pattern demonstrated a statistically significant lower extension CS-ROM compared to those with a Lo breathing pattern. This finding, combined with the positive association between CS-ROM extension and CVA (Table 4), possibly indicates that the adoption of an FHP combined with an increased tension in certain cervicothoracic extensors, sternocleidomastoid, scalene, and anterior chest muscles [41] may lead to the decreased extension of the middle and lower part of the cervical spine in subjects with a Hi breathing pattern. However, we did not identify any similar studies comparing CS-ROM in patients with a Hi vs. Lo breathing pattern.

Furthermore, a trend towards an elevated RR was noted in the Hi compared to the Lo group. While this difference was statistically significant, it is pertinent to consider that the study population comprised individuals with musculoskeletal rather than primary respiratory conditions. Consequently, substantially greater RR values (over 18 breaths/min) would not necessarily be anticipated. Our study findings on RR number align with the findings of Stephen et al. (2022), who investigated the relationship between neck pain and dysfunctional breathing in a cohort study of 49 participants with neck pain and a control group of 49 individuals matched for age and sex [69]. Their results indicated a comparable RR number between participants with (Mean (SD) 15.0 (3.6), Min–Max 9–24 breaths/min) and without neck pain (Mean (SD) 14.7 (3.1), Min–Max 7–20 breaths/min). However, in that study [69], the majority of participants reported low levels of pain intensity. On the contrary, a case series by McLaughlin in 2011 [67], involving twenty-nine outpatients with neck pain (*n* = 12), back pain (*n* = 8), or both (*n* = 9), who had experienced symptoms for a considerable duration (median 24 months, mean 38 months), identified poor breathing profiles characterized by an RR of 20 breaths/min. Also, another study with 29 females with chronic shoulder/neck pain (mean age 48.3 years, mean (SD) pain duration 8 (8) years, and mean (SD) RR of 17 ± 3 breaths/min) and 35 healthy female controls (mean age 41.1 years and mean (SD) RR of 15 ± 3 breaths/min) reported that the between-group difference in RR was statistically significant [70]. In our population, the mean pain duration was much lower (6.39 months on average) than that those two aforementioned studies. Therefore, there may be variability inherent in the breathing responses to pain, influenced by a multitude of factors, encompassing the specific context, the duration of symptoms and the individual emotional reaction to the nociceptive experience [71].

Finally, a statistically significant positive correlation was observed between CVA and CWE-Up (*R* = 0.29, *p* = 0.01), as well as between CVA and extension CS-ROM (*R* = 0.29, *p* = 0.01). In agreement with our own study, the study by Lau et al. (2011) demonstrated a correlation between CWE and FHP [72], possibly denoting a functional inter-connection between the cervical and thoracic spine. However, in contrast to our study, the study by Ozge Solakoglou et al. [73] did not demonstrate any correlation between CWE and FHP in patients with chronic neck pain. Furthermore, CWE correlated significantly with all neck ROM (r = 0.39–0.59) in the study of Wirth et al. 2014 [15] in patients with chronic neck pain, but no such correlations were observed in our study. Differences in population and methodological approaches between studies may have accounted for these disparities.

In summary, the findings of this study suggest that in the context of NSCNP of not particularly prolonged pain duration, an apical breathing pattern (Hi classification) appears to be prevalent. Other possible explanations for participants’ adopting a Hi breathing pattern may have been a sedentary way of life, reduced physical activity [16], smoking habits (frequency and duration), or central-type obesity [74] (that might be restricting diaphragmatic excursion); however, our findings show that there was no effect of self-reported physical activity, smoking frequency, age, and BMI on breathing pattern. Also, the presence of FHP appears to be associated with a limitation in cervical extension, potentially due to an extended upper cervical spine posture coupled with the straightening and flexion of the middle and lower cervical segments. Additionally, FHP appears to be associated both with CWE-Up and CWE-Lw, with the link between those measures perhaps being that the smaller the CVA (the more FHP), the more reduced the CS-ROM (extension and left lateral flexion), allowing for less expansion of the thoracic cage [54].

Among the limitations of our study are that we only measured the within-day test–retest reliability of CS-ROM and CVA using a sample of convenience from a single physical therapy practice. We also did not measure other variables related to the biomechanical/neuromotor control aspects of neck pain, such as neck muscle strength, endurance, proprioception, and thoracic spine ROM, which would have perhaps enhanced the clinical profile of patients with NSCNP, possibly providing some further explanations on the interconnections between variables.

Regarding future avenues of investigation, we are focused on the administration of the Hi-Lo assessment from multiple testing positions, alongside the Lateral Rib Expansion (LRE) test, in the evaluation of breathing pattern dysfunction, to furnish the clinician with a more comprehensive evaluation of respiratory mechanics, as recommended [37]. Furthermore, the reliability of the measures examined could be expanded by examining their between-days inter-examiner reliability, as well as their responsiveness, in future studies. The findings of this study could potentially contribute to the evaluation of additional clinical measures, like neuromuscular control tests. This may assist in identifying knowledge gaps and guide future research endeavors.

## 5. Conclusions

The within-day inter-rater test–retest reliability assessment of CS-ROM with two devices and of the CVA in individuals diagnosed with NSCNP of moderate pain chronicity was excellent, with few errors recorded between testing sessions and for CS-ROM between the two devices.

Reductions in CS-ROM and increases in CVA were age-dependent in the particular NSCNP cohort examined. The significant relationship identified between CVA and CWE, and the numerical superiority of participants classified as having a Hi breathing pattern, possibly signify interconnections between NSCNP and the biomechanical aspect of dysfunctional breathing that need to be evaluated further.

## Figures and Tables

**Table 3:** Descriptive statistics of CS-ROM (six directions), CVA, RR, and CWE for male, female, and all participants.

	Male (*n* = 36)		Female (*n* = 54)		Total (*n* = 90)
	Mean (SD)	Min–Max	Mean (SD)	Min–Max	Mean (SD)
CS-ROM					
Flexion (°)	43.30 (11.65)	24.60–69.73	49.18 (13.93) *	21.17–76.70	46.83 (13.31)
Extension (°)	51.18 (11.62)	29.40–73.40	49.96 (9.00)	29.83–68.47	50.45 (10.09)
R-Side Flexion (°)	36.30 (8.34)	17.90–49.30	37.90 (8.03)	22.43–57.00	37.26 (8.14)
L-Side Flexion (°)	36.32 (7.63)	23.13–55.30	37.84 (7.32)	20.90–53.67	37.23 (7.44)
R Rotation (°)	65.01 (9.75)	42.67–90.50	68.58 (9.41)	48.87–99.83	67.15 (9.66)
L Rotation (°)	64.35 (10.70)	38.6–83.40	69.10 (9.21) *	50.27–89.43	67.20 (10.05)
CVA (°)	45.84 (9.17)	23.50–66.40	49.29 (6.96) *	26.80–62.00	47.91 (8.05)
RR (breaths/min)	15.20 (2.87)	9.17–21.79	15.09 (3.26)	8.59–24.12	15.13 (3.09)
CWE-Up (cm)	4.11 (0.75)	2.50–6.00	3.81 (0.76)	2.00–6.50	3.93 (0.76)
CWE-Lw (cm)	5.35 (1.12)	3.00–7.00	5.05 (1.01)	3.00–7.50	5.17 (1.06)

CS-ROM: cervical spine range of motion, CVA: craniovertebral angle, CWE: chest wall expansion, RR: respiratory rate, Up: upper, Lw: lower, R: right, L: left, cm: centimeters. * *p* < 0.05 (significant level of male–female differences).

**Table 4:** Descriptive statistics of demographics, CS-ROM (six directions), CVA, RR, and CWE for participants with different breathing pattern (Hi vs. Lo).

	Hi (n = 81)	Lo (n = 9)
Sex (male/female)	31/50	5/4
	**Mean (SD)**	**Mean (SD)**
Age (y)	41.53 (11.02)	38.78 (10.27)
Body Mass Index (kg/m^2^)	25.42 (4.43)	25.12 (2.83)
CS-ROM		
Flexion (°)	46.54 (13.57)	49.40 (11.10)
Extension (°)	49.66 (9.74)	57.57 (10.98) *
R-Side Flexion (°)	37.29 (8.15)	37.06 (8.56)
L-Side Flexion (°)	37.32 (7.22)	36.45 (9.71)
R Rotation (°)	66.72 (9.59)	71.07 (9.89)
L Rotation (°)	67.17 (10.19)	67.47 (9.32)
CVA (°)	48.03 (8.10)	46.78 (7.95)
RR (breaths/min)	15.35 (3.03)	13.17 (3.12) *
CWE-Up (cm)	3.86 (0.73)	4.54 (0.80) *
CWE-Lw (cm)	5.10 (1.05)	5.73 (1.05)

CS-ROM: cervical spine range of motion, CVA: craniovertebral angle, CWE: chest wall expansion, RR: respiratory rate, Up: upper, Lw: lower, R: right, L: left, cm: centimeters, y: years, kg: kilograms, m: meters. * *p* < 0.05 (significant level of differences between participants with Hi and Lo breathing pattern).

**Table 5:** Descriptive statistics (mean, SDs) and και reliability indices (ICC_2,3_ for within device, ICC_2,1_ for parallel-forms, SEM, MDC_95%_) for all six CS-ROM movement directions (*n* = 45). With normal font the mean test–retest measures taken with 1-h difference with the KForce Sens^®^ or with the mobile and with italics the two concurrent (parallel-forms) measures between the two devices (Parallel 1 and 2).

Flexion					
Goniometer	Mean (SD) 1	Mean (SD) 2	ICC_2,3_ (95% CI)	SEM (°)	MDC_95%_ (°)
KForce Sens^®^	54.99 (10.57)	54.55 (10.11)	0.98 (0.97–0.99)	1.75	4.85
Mobile	55.31 (10.39)	54.89 (10.05)	0.96 (0.93–0.98)	1.95	5.40
	**KForce Sens^®^**	**Mobile**	**ICC_2,1_ (95% CI)**		
Parallel 1	54.99 (10.57)	55.31 (10.39)	0.99 (0.98–0.99)	0.77	2.14
Parallel 2	54.55 (10.11)	54.89 (10.05)	0.99 (0.98–0.99)	1.05	2.92
**Extension**					
**Goniometer**	**Mean (SD) 1**	**Mean (SD) 2**	**ICC_2,3_ (95% CI)**	**SEM (°)**	**MDC_95%_ (°)**
KForce Sens^®^	51.66 (9.80)	52.23 (9.92)	0.98 (0.97–0.99)	1.82	5.04
Mobile	51.85 (10.23)	51.90 (9.43)	0.90 (0.83–0.95)	3.09	8.56
	**KForce Sens^®^**	**Mobile**	**ICC_2,1_ (95% CI)**		
Parallel 1	51.66 (9.80)	51.85 (10.23)	0.97 (0.95–0.98)	1.74	4.83
Parallel 2	52.23 (9.92)	51.90 (9.43)	0.97 (0.95–0.98)	1.60	4.43
**R-Side Flexion**					
**Goniometer**	**Mean (SD) 1**	**Mean (SD) 2**	**ICC_2,3_ (95% CI)**	**SEM (°)**	**MDC_95%_ (°)**
KForce Sens^®^	36.91 (8.86)	37.26 (8.72)	0.97 (0.95–0.98)	1.44	3.99
Mobile	36.94 (8.76)	37.26 (8.66)	0.99 (0.97–0.99)	1.02	2.82
	**KForce Sens^®^**	**Mobile**	**ICC_2,1_ (95% CI)**		
Parallel 1	36.91 (8.86)	36.94 (8.76)	0.99 (0.97–0.99)	1.07	2.96
Parallel 2	37.26 (8.72)	37.26 (8.66)	0.99 (0.99–0.99)	0.66	1.83
**L-Side Flexion**					
**Goniometer**	**Mean (SD) 1**	**Mean (SD) 2**	**ICC_2,3_ (95% CI)**	**SEM (°)**	**MDC_95%_ (°)**
KForce Sens^®^	38.53 (7.98)	38.39 (8.17)	0.97 (0.95–0.98)	1.33	3.68
Mobile	38.39 (8.08)	38.65 (7.94)	0.98 (0.97–0.99)	1.02	2.82
	**KForce Sens^®^**	**Mobile**	**ICC_2,1_ (95% CI)**		
Parallel 1	38.53 (7.98)	38.39 (8.08)	0.99 (0.98–0.99)	0.81	2.24
Parallel 2	38.39 (8.17)	38.65 (7.94)	0.99 (0.97–0.99)	0.93	2.58
**R Rotation**					
**Goniometer**	**Mean (SD) 1**	**Mean (SD) 2**	**ICC_2,3_ (95% CI)**	**SEM (°)**	**MDC_95%_ (°)**
KForce Sens^®^	69.44 (9.39)	69.70 (8.40)	0.98 (0.96–0.99)	1.88	5.21
Mobile	69.82 (9.37)	70.02 (9.03)	0.98 (0.96–0.99)	1.28	3.54
	**KForce Sens^®^**	**Mobile**	**ICC_2,1_ (95% CI)**		
Parallel 1	69.44 (9.39)	69.82 (9.37)	0.99 (0.98–0.99)	0.75	2.08
Parallel 2	69.70 (8.40)	70.02 (9.03)	0.98 (0.96–0.99)	1.35	3.75
**L Rotation**					
**Goniometer**	**Mean (SD) 1**	**Mean (SD) 2**	**ICC_2,3_ (95% CI)**	**SEM (°)**	**MDC_95%_ (°)**
KForce Sens^®^	70.46 (9.08)	70.93 (9.14)	0.98 (0.97–0.99)	1.73	4.79
Mobile	70.59 (9.22)	70.91 (9.29)	0.98 (0.96–0.99)	1.32	3.66
	**KForce Sens^®^**	**Mobile**	**ICC_2,1_ (95% CI)**		
Parallel 1	70.46 (9.08)	70.59 (9.22)	0.99 (0.99–0.99)	0.54	1.49
Parallel 2	70.93 (9.14)	70.91 (9.29)	0.99 (0.98–0.99)	0.99	2.74

ICC: intraclass correlation coefficient, SEM: standard error of the measurement, MDC: minimum detectable change, ^O^: degrees, R: right, L: left.

**Table 6:** Descriptive statistics (mean, SDs) and και reliability indices (ICC_2,1_, SEM, MDC_95%_) for the assessment of FHP with the CVA (*n* = 45).

	Mean (SD) 1	Mean (SD) 2	ICC_2,3_ (95% CI)	SEM (°)	MDC_95%_ (°)
**CVA**	47.91 (8.41)	48.28 (8.26)	0.97 (0.95–0.98)	1.39	3.85

ICC: intraclass correlation coefficient, SEM: standard error of the measurement, MDC: minimum detectable change, CVA: craniovertebral angle, FHP: forward head posture.

**Table 7:** Correlations (Pearson’s) between patient demographics, CS-ROM (six directions), CVA, respiratory rate, and chest wall expansion (*n* = 90).

		Age	Height	Body Mass	BMI	CVA	RR	CWE-Up	CWE-Lw	Flexion	Extension	R-Side Flexion	L-Side Flexion	R Rotation
Height	R	−0.28 **												
	*p*	0.007												
Body Mass	R	−0.03	0.61 **											
	*p*	*0.80*	*<0.001*											
BMI	R	0.15	0.19	0.90 **										
	*p*	*0.17*	*0.069*	*<0.001*										
CVA	R	−0.21 *	0.04	−0.39 **	−0.52 **									
	*p*	*0.044*	*0.706*	*<0.001*	*<0.001*									
RR	R	−0.08	−0.09	−0.06	−0.04	−0.00								
	*p*	*0.435*	*0.407*	*0.574*	*0.693*	*0.978*								
CWE-Up	R	−0.15	0.28 **	0.09	−0.05	0.29 **	−0.16							
	*p*	*0.145*	*0.008*	*0.406*	*0.659*	*0.005*	*0.120*							
CWE-Lw	R	−0.10	0.18	0.00	−0.10	0.24 *	−0.17	0.79 **						
	*p*	*0.327*	*0.084*	*0.987*	*0.341*	*0.025*	*0.106*	*<0.001*						
Flexion	R	−0.16	−0.17	−0.17	−0.11	0.11	0.14	0.01	−0.02					
	*p*	*0.121*	*0.098*	*0.112*	*0.311*	*0.311*	*0.192*	*0.898*	*0.861*					
Extension	R	−0.29 **	0.20	−0.01	−0.14	0.29 **	0.07	0.06	−0.04	0.13				
	*p*	*0.006*	*0.060*	*0.919*	*0.184*	*0.005*	*0.497*	*0.588*	*0.730*	*0.207*				
R-Side Flexion	R	−0.32 **	0.13	−0.01	−0.09	0.16	−0.11	0.04	0.00	0.10	0.33 **			
	*p*	*0.002*	*0.217*	*0.942*	*0.399*	*0.142*	*0.295*	*0.719*	*0.973*	*0.350*	*0.001*			
L-Side Flexion	R	−0.40 **	0.10	−0.07	−0.15	0.21 *	0.01	0.02	0.04	0.26 *	0.22 *	0.54 **		
	*p*	*<0.001*	*0.328*	*0.528*	*0.147*	*0.049*	*0.934*	*0.835*	*0.694*	*0.013*	*0.038*	*<0.001*		
R Rotation	R	−0.23 *	−0.11	−0.11	−0.08	0.15	0.04	−0.01	−0.04	0.41 **	0.37 **	0.45 **	0.44 **	
	*p*	*0.031*	*0.311*	*0.318*	*0.435*	*0.145*	*0.713*	*0.918*	*0.701*	*<0.001*	*<0.001*	*<0.001*	*<0.001*	
L Rotation	R	−0.28 **	−0.11	−0.25 *	−0.26 *	0.19	0.08	−0.09	−0.08	0.40 **	0.45 **	0.25 *	0.40 **	0.47 **
	*p*	*0.007*	*0.316*	*0.016*	*0.014*	*0.068*	*0.468*	*0.381*	*0.439*	*<0.001*	*<0.001*	*0.016*	*<0.001*	*<0.001*

BMI: body mass index, CVA: craniovertebral angle, CWE: chest wall expansion, RR: respiratory rate, Up: upper, Lw: lower, R: right, L: left. ** *p* < 0.01, * *p* < 0.05.

## Data Availability

The data presented in this study are available on request from the corresponding author. The data are not publicly available due to the applicable data protection law in Greece (Law 4624/2019).

## Abbreviations

The following abbreviations are used in this manuscript:

BMI	Body Mass Index
m	Meters
CS-ROM	Cervical Spine Range of Motion
CVA	Craniovertebral Angle
CWE	Chest Wall Expansion
FHP	Forward Head Posture
ICC	Intraclass Correlation Coefficient
L	Left
Lw	Lower
MDC	Minimum Detectable Change
NSCNP	Non-Specific Chronic Neck Pain
R	Right
RR	Respiratory Rate
SEM	Standard Error of the Measurement
Up	Upper
